# Brain magnetic resonance imaging markers of vigabatrin-associated neurotoxicity during hyperpyrexia-triggered focal status epilepticus: cause or coincidence?

**DOI:** 10.1007/s00247-026-06628-9

**Published:** 2026-04-25

**Authors:** Gabriella Errichiello, Mario Tortora, Adriana Cristofano, Carmela Russo, Antonio Varone

**Affiliations:** 1 Department of Translational Medical Science, Child Neuropsychiatry Unit, University of Naples Federico II, Sergio Pansini st. 5, 80131 Naples, Italy; 2 Department of Neurosciences, Pediatric Neurology Unit, Santobono Childrens Hospital, Naples, Italy; 3 Department of Advanced Biomedical Sciences, Neuroradiology Unit, University of Naples Federico II, Naples, Italy; 4 Department of Neurosciences, Pediatric Neuroradiology Unit, Santobono Childrens Hospital, Naples, Italy; 5 Department of Neurosciences, Pediatric Neurology Unit, Santobono Childrens Hospital, Naples, Italy

**Keywords:** Epileptic spasms, Gamma-aminobutyric acid transaminase inhibitors, Infantile spasms, Magnetic resonance imaging, Neurotoxicity syndromes, Status epilepticus, Vigabatrin

## Abstract

Vigabatrin is an irreversible γ-aminobutyric acid (GABA) transaminase inhibitor and an effective treatment for infantile epileptic spasms syndrome and refractory focal seizures. Although well tolerated and free from major pharmacokinetic interactions, its use is limited by potential adverse effects, including vigabatrin-associated brain magnetic resonance imaging abnormalities. We present a 9-month-old male patient with developmental and epileptic encephalopathy harboring a *POLR2A* gene variant, treated with Vigabatrin (Sabril®, Lundbeck, Copenhagen, Denmark) at 125 mg/kg/day, who presented with febrile focal status epilepticus. Brain magnetic resonance imaging (MRI) showed bilateral and symmetrical involvement of the globi pallidi, subthalamic nuclei, cerebral peduncles, and dorsal pons, characterized by diffusion restriction (diffusion-weighted imaging (DWI) hyperintensity and corresponding apparent diffusion coefficient (ADC) reduction). These MRI abnormalities resolved following discontinuation of vigabatrin. This case highlights the need for clinical awareness and regular neuroimaging during vigabatrin therapy, as MRI abnormalities are often asymptomatic but can occasionally coincide with acute neurological events, especially in the immature brain. Close monitoring is essential to detect early neurotoxicity signs and to consider therapy withdrawal when appropriate, when a direct causal relationship remains uncertain.

## Introduction

Vigabatrin (4-aminohex-5-enoic acid) is an anti-seizure medication and a key treatment for infantile epileptic spasms syndrome and refractory focal seizures. It acts as an irreversible inhibitor of γ-aminobutyric acid (GABA) transaminase, leading to increased GABA levels in the presynaptic terminals of the central nervous system. Vigabatrin also contributes to the attenuation of the glutamate-glutamine cycle, the primary excitatory neurotransmitter system in the central nervous system. Good tolerability and lack of significant interactions with other anti-seizure medications initially made it an ideal first-line option for infantile epileptic spasms syndrome. However, long-term use has been associated with adverse effects such as retinotoxicity (visual field cuts) and neurotoxicity, including often asymptomatic abnormalities detectable on brain magnetic resonance imaging (MRI) [1]. The most affected regions include the globi pallidi, thalami, brainstem, and dentate nuclei [2] (Table 1).

**Table 1:** Main radiological findings of vigabatrin-associated neurotoxicity and their localization across different magnetic resonance imaging sequences

**Magnetic resonance imaging sequences**	Findings	Typical locations
T2-weighted	Bilateral, symmetric hyperintensities	Globus pallidum Dorsal portion of the brainstem, including the medial longitudinal fasciculus Thalamus Dentate nucleus
Diffusion-weighted	Hyperintensity (restricted diffusion)	Same as T2W
Apparent diffusion coefficient map	Mildly decreased ADC values (restricted diffusion)	Same as T2W/DWI
Fluid-attenuated inversion recovery	Variable; may show bilateral, symmetric hyperintensities	Frequently in deep gray matter and the brainstem
Magnetic resonance spectroscopy	Often normal; occasionally increased choline and reduced N-acetylaspartate peak; no lactate peak	Performed in the basal ganglia or thalami

*ADC* apparent diffusion coefficient, *DWI* diffusion-weighted imaging, *FLAIR* fluid-attenuated inversion recovery, *T2W* T2-weighted

Several risk factors have been associated with the development of brain MRI abnormalities, including cryptogenic etiology of infantile spasms, age under 12 months at the start of treatment, concomitant hormone therapy, and high-peak vigabatrin dose (e.g., >165 mg/kg/day) [3]. Between 22% and 32% of pediatric patients treated with vigabatrin for infantile spasms develop these abnormalities [3]. Infants are more susceptible to vigabatrin-induced brain lesions compared to adults due to the immaturity and high metabolic activity of deep brain structures, such as the globi pallidi, thalami, and brainstem, which are actively involved in infantile spasms. Disruption of already dysregulated GABAergic networks during critical periods of neurodevelopment may further increase vulnerability to neurotoxicity [4]. Typically, these lesions are not associated with clinical symptoms, but in rare cases they have been linked to extrapyramidal signs or, even more anecdotally, to acute encephalopathy, especially when combined with hormonal therapy [5].

We present the clinical case of vigabatrin-associated brain MRI abnormalities in a patient treated with vigabatrin (125 mg/kg/day). This case involves a 9-month-old boy with developmental and epileptic encephalopathy, trigonocephaly, a *POLR2A* gene variant, who presented to our attention with focal status epilepticus triggered by hyperpyrexia.

## Case report

**Fig. 1:**
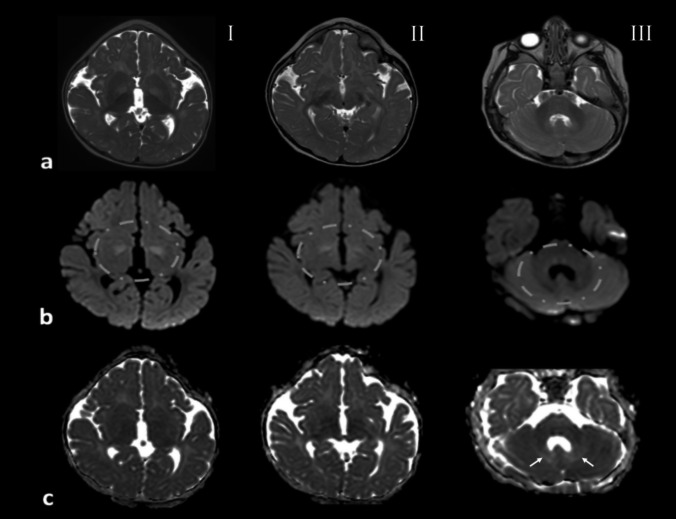
Brain magnetic resonance imaging of a 9-month-old boy with developmental and epileptic encephalopathy, trigonocephaly due to metopic suture synostosis, and a pathogenic *POLR2A* variant, treated with Sabril® (125 mg/kg/day). (*a, b*) Axial T2-weighted turbo spin-echo (*a*) and axial diffusion-weighted (*b*) images without contrast administration demonstrate bilateral and symmetrical hyperintensity (*circles*) involving the globi pallidi (*I*), subthalamic nuclei (*II*), and central tegmental tracts (*III*). There is mild hyperintensity of the dentate nuclei (*arrows*). (*c*) Corresponding axial apparent diffusion coefficient images demonstrate restricted diffusion of all abnormal sites except for the dentate nuclei (*arrows*)

**Fig. 2:**
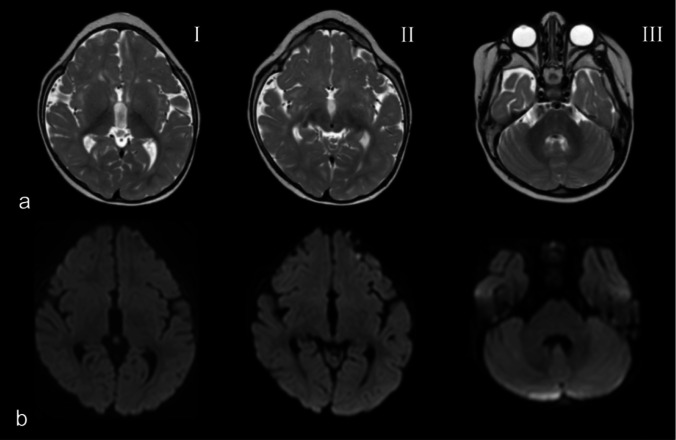
Brain magnetic resonance imaging of the 17-month-old boy with developmental and epileptic encephalopathy, trigonocephaly due to metopic suture synostosis, and a pathogenic *POLR2A* variant, performed 8 months after gradual discontinuation of Sabril® (vigabatrin). (*a, b*) Axial T2-weighted turbo spin-echo (*a*) and axial diffusion-weighted (*b*) images without contrast administration demonstrate normal signal of the globi pallidi (*I*), subthalamic nuclei (*II*), and central tegmental tracts (*III*), with complete resolution of the bilateral and symmetrical deep gray matter and brainstem signal abnormalities, without residual sequelae

The clinical history of this boy began at 22 days of life with neonatal admission for focal seizures, followed by asymmetric epileptic spasms. Trigonocephaly due to metopic suture synostosis was noted. The first video-electroencephalogram showed poorly organized background activity and epileptiform abnormalities, mainly high-voltage slow spike-wave complexes over the left temporo-parieto-occipital evolved to contralateral spread and then formed diffuse bursts interspersed with periods of electro-decremental activity. Initial brain MRI was normal. Treatment with vigabatrin (Sabril®, Lundbeck, Copenhagen, Denmark; titrated up to 125 mg/kg/day) and carbamazepine (Tegretol®, Novartis Farma S.p.A., Origgio, Varese, Italy; 30 mg/kg/day) was initiated. At 9 months, the patient was admitted to our intensive care unit for focal status epilepticus during febrile illness. Brain MRI revealed bilateral, symmetric T2 hyperintensities with restricted diffusion in the globi pallidi, subthalamic nuclei, ventral diencephalon, midbrain, and central tegmental tracts. Mild T2 hyperintensity without diffusion restriction was observed in the dentate nuclei. The spectroscopy revealed increased choline without N-acetylaspartate reduction. Additional findings included delayed myelination, mild corpus callosum hypoplasia, and focal right temporo-parieto-occipital hypoperfusion, possibly postictal (Fig. 1). Meanwhile, clinical evolution included psychomotor delay, axial hypotonia, limb hypertonia, and left-eye strabismus. The whole exome sequencing revealed a *POLR2A* gene variant. The neuroradiological abnormalities resolved following the gradual discontinuation of vigabatrin, as shown by the brain MRI performed 8 months later (Fig. 2).

## Discussion

The MRI findings observed, characterized by bilateral and symmetrical involvement of the globi pallidi, subthalamic nuclei, cerebral peduncles, and dorsal pons, with diffusion restriction (diffusion-weighted imaging (DWI) hyperintensity and corresponding apparent diffusion coefficient (ADC) reduction), prompt a differential diagnosis that includes mitochondrial encephalopathies (such as Leigh syndrome), hypoxic-ischemic injury, metabolic disorders, and drug-induced toxicity. However, the absence of a lactate peak on MR spectroscopy, the clinical context, and the clear radiological improvement following vigabatrin withdrawal strongly support a diagnosis of vigabatrin-associated brain abnormalities.

Although there is no universally accepted guideline recommending the discontinuation of vigabatrin based solely on MRI abnormalities [6], withdrawal should be strongly considered when radiological findings coincide with clinical deterioration. In our case, while a direct causative role of vigabatrin in triggering status epilepticus remains uncertain—only three cases are reported in the literature [7]—both clinical and imaging features markedly improved after drug cessation.

Several authors have described acute neurological deterioration associated with vigabatrin-associated brain MRI abnormalities, including encephalopathy, movement disorders, and in rare cases status epilepticus. For example, Hernández Vega et al. (2014) described a 10-month-old patient with infantile epileptic spasms syndrome who developed life-threatening encephalopathy, characterized by lethargy, movement disorder, and left hemiparesis, after only 7 days of high-dose Sabril® (150 mg/kg/day). MRI revealed deep gray matter abnormalities, and both clinical and radiological features resolved completely after drug discontinuation [8]. Proposed mechanisms for vigabatrin neurotoxicity include excessive GABA accumulation leading to paradoxical excitatory effects in deep brain structures, mitochondrial dysfunction with impaired mitophagy and increased oxidative stress, and selective vulnerability of subcortical regions in the immature brain.

The involvement of the basal ganglia and brainstem, regions consistently affected in vigabatrin-associated brain MRI abnormalities and crucial in seizure modulation, suggests that vigabatrin-induced subcortical dysfunction might contribute to seizure perpetuation in predisposed individuals.

Although vigabatrin-associated brain MRI abnormalities are often asymptomatic and reversible, they may, in select cases, present with acute clinical deterioration. We believe this phenomenon is likely underestimated, particularly in infants, due to its frequent subclinical presentation and spontaneous resolution after treatment discontinuation. In our report, MRI abnormalities were found during focal status epilepticus, raising the uncertain possibility of a contributory role of vigabatrin.

Our experience underscores the importance of integrating radiological findings with clinical evolution and individual susceptibility. When vigabatrin-associated brain MRI abnormalities are suspected, a personalized approach—balancing potential benefits and risks—should guide the decision to discontinue vigabatrin, especially in emergency settings or in the presence of new neurological signs.

## Data Availability

No datasets were generated or analysed during the current study.
